# 

**Published:** 1914-05-02

**Authors:** 


### CONTENTS.

PAGE

**The Problem of Syphilis in Poor-Law Infirmaries** ... ... ... ... ... ... 113

**Colloid Metals in Therapeutics** ... ... ... 114

**Hospital and Institutional News** ... ... ... 115

Hospital Preparations in Belfast—The Volunteer Hospitals in Ulster—The Coming Conference at Newcastle—Honour Where it is Due—Salvarsan in Poor-Law Infirmaries—Winsley Sanatorium and the Charity Commissioners—Interesting the Workpeople — Blackburn "Royal" Infirmary—The Doctors' Wives Association—New Wing at Bristol General Hospital—Should Chairmanships go Round a Committee?—The Infectious Hospital Garden—The Sex Question in Red Cross Work—The Routine of a "Threepenny" Practice—New Assistant Medical Superintendent at Camberwell Infirmary—Wordsworth and a Smallpox Hospital—A Hospital President's Resignation—Queen Alexandra and Infant Mortality—The Clergy and Death Certificates—Hospital Egg Week: May 12 to 19—Unallocated Surplus Funds—Mr. Danckwerts and the Profession—The New Wayland Infirmary—Its Lesson for Poor-Law History—The Misuse of the Term "Infirmary"—The Position of Mexican Drugs—The City Coroner's Pronouncements—The Reporting of Inquests—To Our Readers.

PAGE

**Is Tuberculin Any Use?** ... ... ... ... 121

The King Edward VII. Sanatorium Report.

**Recovery from Sleeping Sickness** ... ... 122

**The Orthopædic Department** ... ... ... 123

VI. The Plaster Room.

**Modern Methods** ... ... ... ... ... ... 125

Colloid Metals.

**Research and Remedies** ... ... ... ... 126

Drinking-Water and Dental Caries—Concerning the First Rib—The Diagnosis of Paternity—Actual Results of the Test.

[See over.]

### CONTENTS—(*continued*).

| | PAGE |
|---|---|
| **Reports on Hospitals of the United Kingdom**— By SIR HENRY BURDETT, K.C.B., K.C.V.O. Ramsgate General Hospital and Seamen's Infirmary. | 127 |
| **The Harrow Cottage Hospital** ... ... ... Its Architecture and its Critics. | 128 |
| **The Institutional Library** ... ... ... ... The Path to Promotion—Speech and Sight for the Dumb and Blind—A Handbook of Hygiene—A Pomp--s "Symposium"—A Credit to British Surgery. | 129 |
| **Round the World** ... ... ... ... ... Fellow-Workers and their Doings. | 131 |
| **Maternity Homes in Industrial Life** ... ... The State and the Environment of Pregnancy. | 132 |
| **The Editor's Letter-Box** ... ... ... ... Salaries of Poor-Law Medical Officers—Non-Panel Men and the "Medical Directory"—Doctor's Precaution against Premature Burial—Infant Hygiene in the Medical Curriculum. | 133 |
| **The Patients' Column** ... ... ... ... ... III. A London and a Provincial General Hospital Experience. | 135 |
| **Personalia** ... ... ... ... ... ... ... | 135 |
| **Doctors' Wills** ... ... ... ... ... ... | 135 |
| **Radium in Deafness** ... ... ... ... ... Hyoscine Injections and a Captious Critic. | 136 |
| **Our Prize Scheme** ... ... ... ... ... | 137 |
| **Buildings Contemplated** ... ... ... ... | 137 |
| **Gifts and Bequests** ... ... ... ... ... | 137 |
| **The Book Market** ... ... ... ... ... | 137 |
| **The National Medical Union (Official)** ... ... The Constitution Committee's Recommendations. | 138 |
| **The Institutional Worker** ... ... (Suppl.) The Chaplain's Department. Waste Materials as a Source of Income. Economy in the Use of Gas. Questions for May. Questions Invited. Employment and Training. | 1 |

